# Insights Into the Processing of Collocations During L2 English Reading: Evidence From Eye Movements

**DOI:** 10.3389/fpsyg.2022.845590

**Published:** 2022-03-30

**Authors:** Hui Li, Kevin B. Paterson, Kayleigh L. Warrington, Xiaolu Wang

**Affiliations:** ^1^School of International Studies, NingboTech University, Ningbo, China; ^2^Department of Neuroscience, Psychology and Behaviour, University of Leicester, Leicester, United Kingdom; ^3^Department of Psychology, Nottingham Trent University, Nottingham, United Kingdom; ^4^School of Foreign Languages, Zhejiang University City College, Hangzhou, China; ^5^School of International Studies, Zhejiang University, Hangzhou, China; ^6^School of Humanities and Communication Arts, Western Sydney University, Penrith, NSW, Australia

**Keywords:** collocation, collocation strength, contextual predictability, eye movements in reading, L2 learners

## Abstract

We report an eye movement experiment that investigates the effects of collocation strength and contextual predictability on the reading of collocative phrases by L2 English readers. Thirty-eight Chinese English as foreign language learners (EFL) read 40 sentences, each including a specific two-word phrase that was either a strong (e.g., *black coffee*) or weak (e.g., *bitter coffee*) adjective-noun collocation and was either highly predictable or unpredictable from the previous sentence context. Eye movement measures showed that L2 reading times for the collocative phrases were sensitive to both collocation strength and contextual predictability. However, an interaction effect between these factors, which appeared relatively late in the eye movement record, additionally revealed that contextual predictability more strongly influenced time spent reading weak compared with strong collocations. This was most likely because the greater familiarity of strong collocations facilitated their integration, even in the absence of strong contextual constraint. We discuss the findings in terms of the value of collocations in second language learning.

## Introduction

Native users of a language often use formulaic language (i.e., recurrent sequences of words) in spoken and written communication. This includes the use of collocations, which are juxtapositions of two or more words that are used together frequently, i.e., phrases, such as “black coffee” or “a quick shower” (Hill, 2000). These phrases are considered to be distinct from other word conjunctions that include compound words, such as “football” and “sunflower,” or hyphenated compounds, such as “machine-made,” where the conjunction of words produces a new or distinctive meaning. Crucially, the use of formulaic expressions, such as collocations, is believed to be a hallmark of linguistic proficiency and therefore, potentially important for the development of linguistic competence by L2 language learners (Cowie, 1998; Wray, 2000; Granger and Bestgen, 2014). This raises the question of whether L2 language learners use knowledge of formulaic language to benefit their production and comprehension. Bearing this issue in mind, with the present experiment, we used measures of eye movements to investigate the L2 processing of collocations during reading comprehension.

The experiment was motivated by the growing evidence that knowledge of collocations can facilitate processes of word recognition in reading. This includes evidence from research that has used variants of the lexical decision task to reveal a recognition advantage for collocations over non-collocative phrases (e.g., Ellis et al., 2009; Durrant and Doherty, 2010; Wolter and Yamashita, 2014). In one such experiment by Durrant and Doherty, participants viewed a series of two-word displays in which a prime word was swiftly followed by a target that was either a real word or a pseudoword. The participant’s task was to indicate whether the target was a real word as quickly and accurately as possible by pressing one of two response keys. The principal finding was that participants were quicker to make lexical decisions when the prime and target word combined to form a collocation (e.g., parish church) rather than a novel phrase (feature church). Such findings, therefore, suggest that readers can use their knowledge of collocations to speed up the process of word recognition.

Other researchers have used eye movement measures to determine whether a similar recognition advantage is observed in normal reading. This approach is based on the assumption that reader’s eye movements are sensitive to how easily linguistic information can be processed so that their gaze dwells for longer on words that are more difficult to recognize (see Rayner, 1998, 2009). There is substantial evidence for this close yoking between eye movements and the process of visual word recognition, including from studies showing that less time is spent looking at words that have a higher frequency of usage in text and so likely to be more familiar to readers (e.g., Inhoff and Rayner, 1986; Rayner et al., 1996). Collocations by their very nature are encountered more often than other phrases and so might be expected to produce similar frequency effects during reading. Consistent with this, an experiment by Vilkaite (2016) showed that collocations, such as “provide information,” are read more quickly compared to non-collocations, such as “compare information.” Similarly, an experiment by Siyanova-Chanturia et al. (2011) showed that binomial phrases, which are a conjunction of words in a specified order (e.g., “bride and groom”), are read more quickly when presented in this order compared to when the word order is reversed (e.g., groom and bride; see Yu et al., 2016, for similar effects for Chinese idioms).

Other research has additionally shown that readers’ eye movements are sensitive to the frequency of collocation usage. Information about a collocation’s frequency of usage can be computed in several ways, i.e., in terms of phrasal frequency, which provide a count of how frequently combinations of words appear together as a phrase (Gries and Ellis, 2015), or in terms of mutual information (i.e., MI, Hunston, 2002), which is a conditionalized count (i.e., a ratio) of how often these words appear together in a phrase rather than separately. Some researchers have also used transitional probabilities, which assess how likely it is that one word will follow another in text, to measure word co-occurrences (McDonald and Shillcock, 2003a,b). An experiment by Sonbul (2015) examined participants’ eye movements when reading sentences containing synonymous phrases that were classified as strong collocations (e.g., fatal mistake), weaker collocations (e.g., awful mistake), or non-collocative phrases (e.g., extreme mistake) based on their relative phrasal frequency and MI. The findings showed that the strong collocations were read fast by L1 readers, that the weaker collocations were read more slowly, and that non-collocations were read most slowly. Such findings suggest that readers’ eye movements are highly sensitive to a collocation’s frequency of written usage.

With the present experiment, we used eye movement measures to investigate whether L2 readers are also sensitive to collocation usage. As might be expected, L2 readers typically have less knowledge of collocations (e.g., Siyanova and Schmitt, 2008) and make more errors using them in both comprehension and production (e.g., Farghal and Obiedat, 1995; Weinert, 1995; Wray, 2002; Nesselhauf, 2003). However, there is evidence to suggest that L2 readers nevertheless develop sensitivity to collocation usage. This includes evidence from phrase judgment tasks showing that L2 readers are faster to recognize collocations when compared to novel phrases with a similar meaning (Wolter and Gyllstad, 2013; see also Ellis et al., 2008). Moreover, eye movement studies with L2 readers show that collocations, such as “provide information,” are read more quickly than similar non-collocative phrases (Vilkaitë and Schmitt, 2017). Similarly, while Sonbul (2015) found that L1 participants could categorize collocations and non-collocations more accurately than L2 readers in an offline rating task, these two groups of readers showed similar sensitivity to the frequency of collocation usage in a subsequent eye movement experiment. The indication, therefore, is that L2 readers can rapidly acquire knowledge about phrasal usage and use this to read more efficiently. We investigated this issue further in the present research using methods from a recent study by Li et al. (2021).

In this recent experiment, L1 participants read either strong collocations, such as “black coffee,” or weaker collocations, such as “bitter coffee,” in sentence contexts where the phrases were either highly predictable or unpredictable from the previous sentence context. This allowed Li et al. (2021) to investigate the relative contribution of frequency of usage and contextual predictability to the processing of collocations. The key finding was that strong collocations were read faster than weak collocations, consistent with Sonbul (2015) and providing further evidence that readers are sensitive to collocation frequency. The findings also showed that phrases were read more quickly in predictable when compared to neutral sentence contexts, consistent with substantial other evidence showing that readers use contextual knowledge to facilitate word recognition (for a review, see Staub, 2015). Crucially, however, this influence of prior sentence context was similar for the strong and weak collocations, suggesting that knowledge about collocation frequency can be used independently of context. Given these findings, our aim was to determine if L2 readers show similar sensitivity to collocation frequency and contextual predictability.

## Materials and Methods

### Participants

Participants were 38 young adults aged 18 − 23 years (*M* = 20, *SD* = 1.2; 24 women) recruited from Zhejiang University. All were native Chinese speakers who reported no history of dyslexia or reading impairment and who had passed the China National College English Test at Band 4. Participants reported the number of years of English language teaching that they had received (*M* = 10.9, range = 10 − 12 years) and self-assessed their English proficiency in terms of reading (*M* = 3.6, range = 3 − 4), listening (*M* = 3.5, range = 2 − 4), writing (*M* = 3.4, range = 2 − 4), speaking (*M* = 3.5, range = 2 − 4), and overall proficiency (*M* = 3.7, range = 3 − 4) using a 7-point Likert scale (where 1 = very poor and 7 = very good). Their proficiency level and self-assessment scores indicated that participants were intermediate L2 learners. It was not possible to use an *a priori* power analysis to estimate the sample size given the lack of closely comparable studies (see Li et al., 2021, for discussion). Accordingly, we used sensitivity analysis to estimate the smallest effect size that might be detected for interaction between collocation strength and context (Lakens, 2021), employing software created by Westfall.^1^ This indicated that the smallest interaction effect that could be detected using our design was in the region of Cohen’s d? = 0.38–0.42, and so a small- to medium-sized effect.

### Stimuli and Design

Stimuli were forty pairs of adjective-noun phrases obtained from the British National Corpus (Burnage and Dunlop, 1992) used as stimuli in Li et al. (2021) experiment. Each pair combined a different adjective with the same noun (e.g., black coffee and bitter coffee). Each pair of adjectives differed in length by no more than one letter and was closely matched across the stimulus set for both letter length (strong collocation, *M* = 6.1, *SD* = 0.3, weak collocation, *M* = 6.1, *SD* = 0.3, *t*(78) = − 0.12, *p* = 0.91), and lexical frequency (strong collocation, *M* = 5.4, *SD* = 0.7, weak collocation, *M* = 4.7, *SD* = 0.1, *t*(78) = 0.93, *p* = 0.36; using frequency scores from the CELEX database, Baayen et al., 1995).

One phrase from each pair was designated a strong collocation and the other as a weaker collocation, using both phrasal frequency and MI scores obtained from the British National Corpus (Burnage and Dunlop, 1992). Phrasal frequency is a measure of how regularly two words are used together as a phrase (e.g., Carrol and Conklin, 2014), and MI scores indicate how often these words appear in the same phrase rather than separately (Gries and Ellis, 2015). Phrases with MI scores of 3 or greater conventionally are considered to be collocations (Hunston, 2002). While both phrases in each stimulus pair had an MI above 3, the strong collocation of each pair had both larger phrasal frequency and larger MI scores when compared to the weak collocation (phrasal frequency, strong collocation, *M* = 343.3, *SD* = 76.5, weak collocation, *M* = 18.0, *SD* = 3.9, *t*(78) = 4.25, *p* < 0.001; MI, strong collocation, *M* = 8.5, *SD* = 0.3, weak collocation, *M* = 4.3, *SD* = 0.1, *t*(78) = 13.41, *p* = 0.015). Ten Chinese L2 learners of English who did not take part in the eye movement experiment gave familiarity ratings using a 5-point scale (where 1 = not familiar and 5 = very familiar). Scores for all collocations were above 3 (strong collocations, *M* = 4.2, *SD* = 0.1, weak collocations, *M* = 4.1, *SD* = 0.1), showing that all the collocations were considered to be familiar expressions.

**FIGURE 1:**
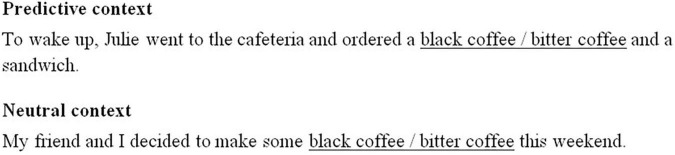
An example stimulus. Collocations are shown underlined with the alternative strong and weak collocations separated using a slash. These stimuli were shown as normal in the experiment, including either strong or weak collocation.

These phrases were presented to participants in two sentence contexts: a neutral context in which both phrases were unpredictable, and a predictable context in which information relevant to the collocation was anticipated. Contextual predictability was assessed by Li et al. (2021) using a cloze task with native English readers. Participants were presented with sentences truncated immediately before the collocation and asked to provide a written continuation. Predictability was assessed by examining if the continuation included the collocation (e.g., black coffee and bitter coffee) or a related concept, such as “cup of coffee” or “espresso.” The collocation was considered predictable if more than 50% of responses were of this type. Collocations were considered unpredictable if less than 20% of responses were of this type. Note that it is more common in the literature to assess the predictability of a specific word (see, e.g., Staub, 2015), whereas we were more interested in determining whether the more general concept was predicted from the prior discourse context. Note also that the MI and frequency scores for the collocations indicate that the adjective and nouns co-occur more often for strong than weak collocations. We would therefore anticipate the noun to be more predictable in a strong than weak collocation, and this would contribute to the shorter reading times for strong collocations observed in previous research. Li et al. (2021) also assessed the naturalness of the stimuli with native English speakers. No differences were observed for sentences containing strong and weak collocations, suggesting that the phrases were similarly acceptable in predictable contexts (strong collocations, *M* = 4.1, *SD* = 0.3, weak collocations, *M* = 4.1, *SD* = 0.3) and neutral contexts (strong collocations, *M* = 4.0, *SD* = 0.4, weak collocations, *M* = 4.0, *SD* = 0.3). The length of sentences ranged from 8 to 19 words (*M* = 12.9, *SD* = 2.41) and the collocation always appeared at the center of the sentence. An example of the stimuli is shown in Figure 1.

The sentence stimuli were divided into two lists. Each of these lists included half of the predictable sentence frames and half of the neutral sentence frames. One of each collocation pair was presented in a neutral sentence frame and the other in a predictable sentence frame in one list, and this allocation of collocation to sentence frame was reversed for the other list. This approach ensured that participants viewed an equal number of collocations in neutral and predictable contexts. The 80 experimental sentences were mixed with 50 filler sentences, and the lists each began with 8 practice sentences. Participants were assigned randomly to one list. Each participant read 138 sentences. The experiment manipulated the within-participants variables of context (predictable, neutral) and collocation strength (strong, weak).

### Apparatus and Procedure

An EyeLink 1000 Plus Eye Tracker was used to record eye gaze from the participant’s right eye location each millisecond during binocular reading. Stimuli were presented on a 19-inch monitor in 14-point Courier New font, as black text on a light gray background. At a 63-cm viewing distance, a 4-letter word would subtend approximately 1^°^. Text displays were therefore of normal size for reading.

Participants were tested individually. Each participant was instructed to read normally for comprehension. At the start of the experiment, the eye-tracker was calibrated to the participant’s right eye movements using a three-point horizontal procedure (ensuring < 0.35 degrees of spatial error). At the start of each trial, a fixation cross was shown on the left side of the screen. Shortly after the participant fixated on this location, the cross disappeared and a sentence was presented with its first letter appearing in place of the cross. Participants pressed the response button once they had finished reading and the sentence disappeared, replaced by a comprehension question requiring a yes/no response on 25% of trials. The participant responded to the question by pressing one of two response buttons. Calibration accuracy was checked prior to each trial and the eye-tracker was recalibrated as required to maintain high spatial resolution. The experiment took about 40 min for each participant.

## Results

Accurately answering the comprehension questions that followed sentence displays was generally high (*M* = 90%) and was not significantly different across experimental conditions (*p*s > 0.1), indicating that the L2 readers could understand the sentences well. Before analyzing the data, short fixations (< 80 ms) were pooled with adjacent fixations, and then fixations less than 80 ms or more than 1,000 ms were removed following a standard procedure. In addition, fixations greater than 2.5 *SD* from each participant’s condition mean were deleted as outliers (accounting for < 3% of data).

The remaining data were analyzed using linear mixed-effects models (LMEMs, Baayen et al., 2008) using the R statistical programming environment (R Core Team, 2019) and the lme4 statistical package (version 1.1-26; Bates et al., 2012). For all analyses, participants and stimuli were treated as crossed random effects, and context and collocation strength were treated as fixed factors. The “contr.sdif” function in the MASS package was used to implement contrasts that compared the different levels of the fixed factors (Venables and Ripley, 2002). A full random structure model was used when possible (Barr et al., 2013). However, if the full model did not converge, its random structure was trimmed until it did converge successfully (starting with removing correlations between factors, then interactions). Significant interactions were investigated further using the “emmeans” package (Lenth et al., 2018). For all analyses, effects, where t/z > 1.96, were considered to be statistically significant (see, e.g., Baayen, 2008).

Eye movement measures are reported for the collocation. These included first-pass measures for the collocation (i.e., its initial processing before a saccade was made to the right of the collocation or a regression was made to its left), which were informative about factors affecting its initial processing. We examined first-pass reading time (FPRT, summed first-pass fixations within a region) and regressions-out (RO, probability of a regressive eye movement from a region) as measures of first-pass processing, and regression-path duration (RPD, summed fixations starting from the first fixation within a region, and following a regression, prior to a fixation to its right; see Liversedge et al., 1998), total reading time (TRT, summed fixations within a region), and regressions-in (RI, probability of a regressive eye movement back into a region), as measures that are informative about the later processing of the collocation. Note that skipping rates (i.e., the probability of not fixating the collocation during first-pass reading) were very low, even for collocation nouns. Specifically, for the collocation nouns, in predictable contexts we observed skipping rates of 8% (*SD* = 1%) for strong collocations and 6% (*SD* = 1%) for weak collocations, and for neutral contexts, we observed noun skipping rates of 4% (*SD* = 1%) for strong collocations and 5% (*SD* = 1%) for weak collocations.

**FIGURE 2:**
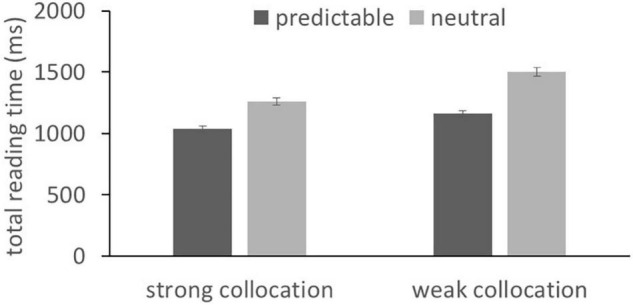
Interactions between collocation strength and contextual predictability in total reading times for the collocation. Error bars correspond to the Standard Error of the Mean.

The means of the eye movement measures are reported in Table 1 and the corresponding statistical results are reported in Table 2. Main effects of context predictability were obtained in all measures, with the exception of RO. These were due to faster reading times and fewer regressions back to collocations in predictable than neutral contexts. In addition, the main effects of collocation strength were obtained for all measures with the exception of RO. These were due to faster reading times and fewer regressions back for the strong relative to the weak collocations. Finally, a significant interaction between context and collocation strength was observed in TRTs (as shown in Figure 2). This reflected a larger predictability effect (i.e., shorter reading times in predictable compared to neutral contexts) for weak relative to strong collocations. Ideally, we would compare the interaction’s effect size against the minimal detectable effect size we report in the Method. However, calculating effect size (and using this to derive statistical power) is notoriously non-trivial for complex LMEMs with more than one fixed effect and two random effects (see, e.g., Westfall et al., 2014; Brysbaert and Stevens, 2018; Kumle et al., 2021). This was therefore not possible to compute for the interaction effect, although we note that, having computed the minimal detectable effect size, our design is relatively well-powered to detect small- to medium-sized effects.

**TABLE 1:** Eye movements for the collocation.

	Predictable context		Neutral context	
Measure	Strong collocation	Weak collocation	Strong collocation	Weak collocation
First-pass reading time (ms)	681 (14)	721 (14)	750 (15)	784 (16)
Regressions-out (%)	17 (1)	18 (1)	19 (1)	19 (1)
Regression-path duration (ms)	872 (18)	918 (18)	968 (20)	1,030 (22)
Total reading time (ms)	1,036 (24)	1,161 (27)	1,260 (29)	1,502 (34)
Regressions-in (%)	25 (2)	33 (2)	38 (2)	43 (2)

*The SE of the mean is shown in parentheses.*

**TABLE 2:** Summary statistics for the collocation.

Factor	Statistic	FPRT	RPD	TRT	RI	RO
Intercept	β	733.54	947.94	1244.19	–0.74	–1.63
(global mean)	SE	32.67	46.89	79.8	0.13	0.12
	t/z	57.07	20.21	15.59	–5.78	–13.47
Context	β	–63.75	–104.33	–279.04	–0.59	0.1
(predictable-neutral)	SE	12.74	16.74	22.55	0.09	0.13
	t/z	–5.00*	–6.23*	–12.37*	–7.18*	–1.37
Collocation	β	36.41	56.23	182.92	0.36	0.01
(weak-strong)	SE	12.74	24.41	22.56	0.09	0.1
	t/z	2.86*	2.30*	8.11*	4.44*	0.1
Context × Collocation	β	5.25	–19.23	–116.38	0.15	0.05
	SE	25.61	33.55	45.32	0.16	0.19
	t/z	0.21	–0.57	–2.57*	0.9	0.28

Asterisks indicate statistically significant effects, p < 0.05. FPRT, first-pass reading time; RPD, regression-path duration; TRT, total reading time; RO, regressions-out; RI, regressions-in. Model for FPR and TRT, lmer (depvar − context*type_coll + (1| pp) + (1| stim), data = data); Model for RPD: lmer (depvar − context*type_coll + (1 + type_coll| pp) + (1| stim), data = data); Model for RI and RO: glmer(depvar ∼ context*type_coll + (1| pp) + (1| stim), data = data, family = binomial).

## Discussion

The present experiment was conducted to shed light on the use of knowledge about collocation frequency and contextual predictability in L2 reading. The Chinese L2 English readers who took part had relatively long reading times and high rates of regression (collocation reading times averaged 680 ms or more, and collocation regression rates averaged 25% or more), consistent with their status as intermediate L2 readers. Despite this, the eye movement behavior of the L2 readers showed sensitivity to both the frequency of usage of collocations and their predictability from the prior sentence context.

We investigated the effects of collocation frequency by examining eye movements for collocations that were categorized as either strong or weak, depending on their frequency of written usage (as defined using phrasal frequency and MI). The results showed that less time was spent gazing at strong as compared to weak collocations; while, there were larger regression effects for the weak collocations. These effects were obtained in both measures of first-pass and later processing. The presence of effects in measures sensitive to the first-pass processing of the collocation suggests that collocation strength influenced how readily these phrases could be recognized by the L2 readers, with faster recognition for the stronger, i.e., higher frequency and collocations. This aspect of our findings is in line with other evidence from L1 and L2 readers showing that collocation frequency can influence the first-pass processing of a phrase (e.g., Sonbul, 2015; Li et al., 2021). Such findings are important as they imply that frequency of exposure to a specific collocation influences how information about this phrase is encoded in the mental lexicon and used subsequently to facilitate recognition of these expressions. The finding that the processing advantage for strong over weak collocations was also observed in later measures of processing (i.e., regressions back to the collocation) suggests that this processing advantage persists until later during processing and may affect how readily the phrases can be integrated as part of the sentence meaning. This contrasts with the findings from Sonbul (2015), who observed the effects of collocation frequency only in first-pass reading. However, similar effects were reported for L1 readers by Li et al. (2021) using the same stimuli, and so this late effect is unlikely to be unique to L2 readers. We note, however, that we did not test this formally, by directly comparing L2 data from the current experiment with L1 data from this previous research, as these two groups may differ in other respects.

We additionally investigated the effects of contextual predictability on phrase processing by placing the collocations in either a predictive or neutral sentence context. The results showed that readers spent less time at the collocations when they were predicted by the context and with increased regression rates when the collocations were unpredicted. This pattern of findings is consistent with a wealth of other research showing that readers make use of their knowledge of the prior linguistic context to guide the processing of new linguistic information (for a review, see Staub, 2015). Moreover, our findings are in line with the findings from other studies of L2 readers showing that eye movements are affected by both the frequency of usage and contextual predictability of words (e.g., Whitford and Titone, 2017; Mor and Prior, 2021). The predictability effects we observed were emerged in measures of first-pass processing and were also observed in later measures of processing. It, therefore, appears that L2 readers can make rapid use of their knowledge of the prior context to guide the recognition of collocative phrases.

Crucially, the present experimental design also allowed us to assess the conjoint effects of collocation strength and contextual predictability on the performance of the L2 readers. These variables were shown to interactively influence the processing of the collocations. This interaction effect was emerged in relatively late eye movement measure, in TRTs for the collocation. This effect (see Figure 2) was due to larger predictability effects (i.e., longer reading times in neutral vs. predictive contexts) for the weak when compared to strong collocations. As this effect was emerged relatively late in processing, it seems likely that it reflects the integration of the collocation with the sentence context rather than an influence on word recognition processes. The pattern of effects suggests that, in the absence of a constraining context, L2 readers experienced greater difficulty when attempting to integrate the weaker collocations with the sentence. This interaction between predictability and collocation strength stands in contrast with evidence showing that these two factors independently influence L1 readers’ eye movements (Li et al., 2021). Again, while this would be interesting to test formally, by comparing the performance of the two groups, we chose not to do this because of likely uncontrolled group differences. We nevertheless consider that these findings might be suggestive of L2 readers who had difficulty in integrating words with the context in the absence of strong contextual or lexical cues.

Taken together, the present findings add to the growing evidence that knowledge about collocations can influence the eye movements of L1 and L2 readers, and therefore that knowledge about formulaic language has an important influence on reading. Other evidence for such effects comes from studies of idioms (e.g., kick the bucket, Underwood et al., 2004; Conklin and Schmitt, 2008), binomial phrases (e.g., salt and pepper, Siyanova-Chanturia et al., 2011) and collocations (e.g., Sonbul, 2015; Vilkaite, 2016; Vilkaitë and Schmitt, 2017; Li et al., 2021). Among these studies, Sonbul also showed that L1 and L2 readers are sensitive to the frequency of usage of collocations, providing evidence that eye movement behavior is sensitive to the co-occurrence of words as a phrase. Such findings are important to our understanding of what information is accumulated through reading experience. It also challenges a key assumption made by current models of reading (e.g., the E-Z Reader model; Reichle et al., 1998, 2003) which assumes that the language processor employs information relating to the frequency of individual words but not phrases. Research with formulaic language, and collocations, in particular, reveal that L1 and L2 readers are highly sensitive to the frequency with which words occur together in phrases. The present findings additionally show that the integration of phrases with context is a product of the reader’s knowledge of collocations, such that better-known collocations (which will have a higher frequency of usage), are more readily integrated in the absence of contextual constraint. Moreover, the fact that such effects are detectable in eye movements reveals that such knowledge is rapidly brought to bear on the reading process by L1 and L2 readers. Further work is nevertheless needed to understand mechanisms underlying the acquisition and usage of this knowledge and the extent to which differences are observed as a function of reading skills. This will include understanding how similarity (and dissimilarity) between L1 and L2 collocations might affect processing, including for example whether an equivalence between L1 and L2 collocations might contribute to frequency effects.

## Data Availability Statement

A stimulus list, eye movement datafiles and analysis scripts are published on Figshare with the following DOI: 10.25392/leicester.data.17693798. Files are available from to publication from the following link: https://figshare.com/s/80b83e946d1854f46aee.

## Ethics Statement

The studies involving human participants were reviewed and approved by Zhejiang University. The patients/participants provided their written informed consent to participate in this study.

## Author Contributions

HL, XW, and KP designed the experiment. HL collected the data. HL and KW analyzed the data. HL and KP wrote the manuscript. XW gave critical comments. All authors contributed to the article and approved the submitted version.

## Conflict of Interest

The authors declare that the research was conducted in the absence of any commercial or financial relationships that could be construed as a potential conflict of interest.

## Publisher’s Note

All claims expressed in this article are solely those of the authors and do not necessarily represent those of their affiliated organizations, or those of the publisher, the editors and the reviewers. Any product that may be evaluated in this article, or claim that may be made by its manufacturer, is not guaranteed or endorsed by the publisher.

## Footnotes

1
http://jakewestfall.org/
