# Supplementary material for: Insights Into the Processing of Collocations During L2 English Reading: Evidence From Eye Movements
Source: Front Psychol. 2022 Mar 30;13:845590. doi: 10.3389/fpsyg.2022.845590 (PMC9005965; doi:10.3389/fpsyg.2022.845590)
Supplement: Supplementary file 3 [file Table_3.DOCX]

**Experimental Stimuli**

The following is the stimulus set used in an experiment investigating effects of predictive versus neutral contexts on eye movements for strong and weak collocation. The strong and weak contexts are shown in bold, separated by a slash, in each context.

1.

Predictive context

Emotionally resilient people will not fall apart under **intense pressure/extreme pressure** at the workplace.

Neutral context

It didn’t take long for everyone to find out about the **intense pressure/extreme pressure** that John was under.

2.

Predictive context

To help identify the robber, the eyewitness provided a **detailed description/specific description** of his appearance.

Neutral context

On the news, the shopkeeper saw a **detailed description/specific description** of the robber’s appearance.

3.

Predictive context

By following directions on the webpage and using a map, he found the **exact location/fixed location** of the store.

Neutral context

Jessica was surprised that her friend did not mention the **exact location/fixed location**of the museum.

4.

Predictive context

Under the Data Protection Act, patients have the right to see their **medical records/previous records** at any time.

Neutral context

The boss announced to all the workers that the **medical records/previous records** would be made publicly available.

5.

Predictive context

I would like a job in the UK but my poor English is a **major problem/severe problem** in achieving my goal.

Neutral context

For many young people in the world, the **major problem/severe problem** to become independent is buying a house.

6.

Predictive context

To be a good judge, one has to draw a clear **dividing line/boundary line** between good and evil.

Neutral context

It is difficult in some sectors to define a **dividing line/boundary line** between the town and the countryside.

7.

Predictive context

At rush hour, the busy road is always jammed by **heavy traffic/slow traffic** so it is difficult to get home.

Neutral context

The newcomer is not used to the **heavy traffic/slow traffic** in the big city during the rush hour.

8.

Predictive context

Leicester’s different ethnicities, food and dialects reflect the **cultural diversity/wonderful diversity** of this city.

Neutral context

My older sister thinks that there is a lot of **cultural diversity/wonderful diversity** in this famous historic city.

9.

Predictive context

Young drivers who have less experience are more likely to make **fatal mistakes/awful mistakes** while driving.

Neutral context

Barbara took a deep breath and explained how the **fatal mistakes/awful mistakes** resulted in the company’s collapse.

10.

Predictive context

The doctor told me that nail biting and nose picking were **bad habits/odd habits** and they could harm my health.

Neutral context

My mum told me that I should not allow the **bad habits/odd habits** to take over my life.

11.

Predictive context

In a healthy relationship, both partners will have **mutual respect/genuine respect** for one another.

Neutral context

Recent research demonstrates the importance of **mutual respect/genuine respect** in the workplace.

12.

Predictive context

After the earthquake, many buildings have suffered **extensive damage/terrible damage** in the historic city.

Neutral context

The events in the famous city has caused **extensive damage/terrible damage** to many buildings.

13.

Predictive context

The street signs were shaking and the branches of the trees broke due to the **strong wind/fierce wind** last night.

Neutral context

It was right around this time of my life that a **strong wind/fierce wind** began to blow.

14.

Predictive context

Mary wanted a small birthday party and so invited only her **close friends/real friends** and a few relatives.

Neutral context

Emily told everyone how she had learned that **close friends/real friends** are really important.

15.

Predictive context

With the rising cost of housing, many tenants must accept poor **living conditions/current conditions** in the cities.

Neutral context

My parents always tell me and my brother that our **living conditions/current conditions** are better than in the past.

16.

Predictive context

The Office for National Data is the largest independent producer of **official statistics/accurate statistics** in the UK.

Neutral context

More than thirty people died in the accident according to the **official statistics/accurate statistics** released today.

17.

Predictive context

Japan must import copper, rubber, and other **raw materials/key materials** from other countries.

Neutral context

Jim couldn’t believe his luck when he saw that the **raw materials/key materials** were on sale.

18.

Predictive context

People rely on radio and other types of **mass media/local media** for information about the outside world.

Neutral context

There can be little doubt that the **mass media/local media** are a vital part of the political system.

19.

Predictive context

During the monsoon season, many roads were flooded after **heavy rain/steady rain** and needed to be closed.

Neutral context

It is very hard for people to enjoy **heavy rain/steady rain** in winter because it is often cold.

20.

Predictive context

The popular Christmas fireworks display attracted **huge crowds/busy crowds** of people.

Neutral context

Traditionally, it is not very common to see **huge crowds/busy crowds** of people at the park.

21.

Predictive context

Large amount of CO2 emissions cause **global warming/rapid warming** which will disrupt the world's climate.

Neutral context

Nowadays, there is a great deal of talk of **global warming/rapid warming** and its effects on people's life.

22.

Predictive context

Lizards, Rhinoceros and other **wild animals/rare animals** adapt badly to zoo life.

Neutral context

Every year, the lives of many **wild animals/rare animals** are destroyed by people.

23.

Predictive context

British actress Emma Watson will play a **leading role/primary role** in the new film.

Neutral context

The Princess of Wales is to take a **leading role/primary role**in a homeless charity.

24.

Predictive context

To wake up, Julie went to the café and ordered a strong **black coffee/bitter coffee** and a sandwich.

Neutral context

My friend and I decided to make some **black coffee/bitter coffee** this weekend.

25.

Predictive context

The most destructive, inhumane instruments of war are **nuclear weapons/military weapons** which should be prohibited.

Neutral context

In the early years of the 20th century, the numbers of **nuclear weapons/military weapons** in the US grew quickly.

26.

Predictive context

Social scientists have identified poverty as one of the **underlying causes/significant causes** of crime.

Neutral context

At present, people are still not very clear about the **underlying causes/significant causes** of stress.

27.

Predictive context

Animals use body language instead of **verbal communication/written communication** to exchange messages with one another.

Neutral context

It is well known that **verbal communication/written communication** is the central concern for the study of language.

28.

Predictive context

The internet has significantly changed the **everyday life/ordinary life** of people.

Neutral context

People wish to find out more about **everyday life/ordinary life** in the Indus Valley.

29.

Predictive context

The UK government's mass surveillance programme violated **human rights/basic rights** and had no real safeguards.

Neutral context

Professor David Collis will deliver a lecture about **human rights/basic rights** at our university next week.

30.

Predictive context

A good marriage requires a **joint effort/real effort** of husband and wife.

Neutral context

This wonderful party was a **joint effort/real effort** of my friends and I.

31.

Predictive context

The noisy kids put the grumpy old man in a **bad mood/dark mood** and made him shout loudly.

Neutral context

All the group agreed that having a **bad mood/dark mood** is not good for the office atmosphere.

32.

Predictive context

Economic hardship and stressful life events increase the risk of poor **mental health/general health** of people.

Neutral context

Tom watched a television programme about how **mental health/general health** is a big problem in the UK.

33.

Predictive context

His poor judgement suggested he lacked the **common sense/normal sense** most people had.

Neutral context

I believe William had much more **common sense/normal sense** than you realized.

34.

Predictive context

A good way of traffic calming is to get more people to use **public transport/modern transport** on working days.

Neutral context

It would be much more difficult with the lack of **public transport/modern transport** to get to the remote areas.

35.

Predictive context

They insisted that they were not the criminals but rather the **innocent victims/ultimate victims** of this crime.

Neutral context

It was obvious that Bill and Emily were **innocent victims/ultimate victims** of the terrorist attack.

36.

Predictive context

When you're stressed, people often advise you to take a **deep breath/slow breath** to calm down.

Neutral context

It is strange that the pain becomes worse when I am taking a **deep breath/slow breath** or lying flat.

37.

Predictive context

In hard times, the faith of even a **firm believer/true believer** can be tested.

Neutral context

The woman was a **firm believer/true believer** that she was following the will of God.

38.

Predictive context

Last year, the sales of the company rose 12% and its **net profit/pure profit** rose 28%.

Neutral context

We were happy when we found out that the **net profit/pure profit** rose by 12% last year.

39.

Predictive context

To manage your weight, you should control your calorie intake by eating a **balanced diet/regular diet** every day.

Neutral context

Many of us don't know what a **balanced diet/regular diet** is and how important it is to keep healthy.

40.

Predictive context

Infectious diseases can be spread by **direct contact/slight contact** between people.

Neutral context

I don't know whether Mary had **direct contact/slight contact** with customers or not.
